# Use of black pepper oil in growing-quail diets and its impact on growth, carcass measurements, intestinal microbiota, and blood chemistry

**DOI:** 10.5194/aab-67-445-2024

**Published:** 2024-09-24

**Authors:** Fayiz M. Reda, Ayman S. Salah, Youssef A. Attia, Rashed A. Alhotan, Mohamed A. Mahmoud, Alessandro Di Cerbo, Mahmoud Alagawany

**Affiliations:** 1 Poultry Department, Faculty of Agriculture, Zagazig University, Zagazig 44511, Egypt; 2 Department of Animal Nutrition and Clinical Nutrition, Faculty of Veterinary Medicine, New Valley University, Kharga, New Valley, Egypt; 3 Department of Animal and Poultry Production, Faculty of Agriculture, Damanhour, Damanhour 22516, Egypt; 4 Department of Animal Production, College of Food and Agricultural Sciences, King Saud University, Riyadh, Saudi Arabia; 5 Department of Physiology, Faculty of Veterinary Medicine, New Valley University, Kharga, New Valley, Egypt; 6 School of Biosciences and Veterinary Medicine, University of Camerino, 62024, Matelica, Italy

## Abstract

Supplements derived from plants are utilized to maintain and promote the growth efficiency of animals. The use of black pepper oil (BPO) has recently generated significant scientific interest, primarily because of its potential beneficial effects on both humans and animals. The goal of the current study was to determine how dietary BPO supplementation affected growth performance, characteristics of growing quails' immunity, antioxidant status, and carcass yield. A total of 240 growing quails (1 week old) were divided into five equal groups, each with 36 birds (four replicates of 12 each). A basal diet containing no BPO (0 $g\;{\mathrm{kg}}^{-1}$ ) was given to the first group, and the second, third, and fourth groups were given a diet that was supplemented with BPO (0.4, 0.8, 1.2, and 1.6 $g\;{\mathrm{kg}}^{-1}$ , respectively). In comparison to a control group, quails' diets that were supplemented with 0.8 $g\;\mathrm{BPO}\;{\mathrm{kg}}^{-1}$ showed improvements in final live body weight, body weight gain, and feed conversion ratio of 10.68 %, 12.6 %, and 18.2 %, respectively. During the whole study period (1 to 5 weeks), quails fed diets with 0.8 gBPO consumed less feed than the other groups and control. Due to BPO treatment, there were no statistically significant changes in any of the carcass parameters. BPO-supplemented groups had significantly elevated plasma levels of albumin and globulin than control groups ( P < 0.05), but the albumin/globulin ratio was reported to be significantly decreased ( P < 0.05) in birds supplemented with diets containing BPO compared to the control group. When compared to the control, the liver enzyme activity (aspartate transaminase (AST) and alanine transaminase (ALT)) in blood plasma was reported to be significantly increased in the quails given 0.4 and 0.8 $g\;\mathrm{BPO}\;{\mathrm{kg}}^{-1}$ . Glutathione and catalase activities were significantly higher in the group given diets supplemented with BPO (1.2 $g\;{\mathrm{kg}}^{-1}$ ) than they were in the control group. In comparison to the control, the supplementation of BPO in the diets of quail significantly enhanced ( P < 0.05) the lipid profile in the plasma, moreover decreasing the caecal content pH ( P < 0.05). In comparison to the control, the populations of lactobacilli, coliform, *Salmonella* spp., and *Escherichia coli* in the caecum significantly decreased in the BPO-supplemented groups ( P < 0.05). In conclusion, dietary BPO supplementation in Japanese quails' diet can boost growth performance and antioxidant indices, enhance lipid profile and carcass traits, and reduce intestinal infections.

## 1 Introduction

An essential component of the existing research on phytobiotic uses in poultry diets is using herbs and spices as feed supplements (Al-Kassie et al., 2012; Puvača et al., 2015; Saeed et al., 2018). These supplements have been effectively incorporated into the diet to enhance the health of chickens and their welfare (Alagawany et al., 2019; Elwan et al., 2019; Salah et al., 2021; Moharreri et al., 2022).

Phytogenic feed supplements are substances derived from plants that are added to animal feed to improve growth performance (Vakili et al., 2022). In addition to increasing antioxidant, antiviral, anthelmintic, and antibacterial properties, active compounds in spices, herbs, and extracted oils can enhance the secretion of digestive enzymes, boost feed intake, and activate the immune system (Dalle Zotte et al., 2016). Because of its seductive smell, typically spicy flavor, and tingly sensation, black pepper, BP (*Piper nigrum* L.), is a well-known spice (Srinivasan, 2007). Black pepper oil (BPO) is extracted by steam-distilling the dried fruit of the BP plant (*Piper nigrum* L.). It is frequently utilized as a flavoring substance in food products, cosmetics, and aromatherapy. The possible health advantages of BPO for animals, notably Japanese quail, have gained more attention in recent years. Enhanced digestion, anti-inflammatory properties, antibacterial properties, enhanced respiratory health, and stress reduction are a few advantages of black pepper oil for quail that have been previously highlighted. The majority of the studies have either been carried out in vitro or on other poultry species, therefore it is crucial to keep in mind that there has only been little study on the effects of black pepper oil, especially in quail. Black pepper increases nutrient digestive efficiency.

Black pepper oil contains a compound called piperine, which has important nutritional and medicinal effects on the neuromuscular system, It can also promote and speed up fat thermogenesis, improve digestion and absorption, and improve nutrition (Malini et al., 1999). With regard to black pepper's ability to effectively stimulate the gut and enhance digestive output, it has been used extensively for bronchitis, cancer, and stomach problems. When medicinal herbs were included in poultry diets, the body weight gain and feed conversion ratio improved (Iqbal et al., 2011). Compared to chili, BP has been discovered to have antioxidant benefits and an anticarcinogenic impact (Nalini et al., 2006).

In traditional Indian families, black pepper has been utilized as a common treatment for many of human diseases (Moorthy et al., 2009). It suppresses lipid peroxidation, inhibits induced oxidative stress, and destroyed various free radicals such as superoxide and hydroxyl radicals (Weiner, 1994). In total, 80 % of the world's population still relies on medicinal and herbal plants and natural remedies for the majority of their healthcare requirements.

Black pepper has been used medicinally for a variety of illnesses, including the treatment of piles and worms. It also has antibacterial effects, antifungal, antidepressant, antidiarrheal, and inflammatory properties. It also has antitumor, antioxidant, antispasmodic, and antipyretic properties (Ahmad et al., 2012; Islam et al., 2015). Terpenoids, the main components of black pepper essential oil (BPEO), have been found to have possible antibacterial activity (Menon et al., 2003; Zengin and Baysal, 2014). It is predicted that including BPO in the diet will have positive benefits on quail that are in the growing period. Therefore, the goal of the present study was to investigate the effects of dietary supplementation of various BPO levels on growth performance, antioxidant indices and immunity, lipid profile, cecal microbiota, and carcass traits of the growing quail.

## 2 Materials and methods

### 2.1 Animals, experimental design, and diets

A total of 240 1-week-old Japanese growing quail chicks, with an average initial weight of 29.65 ± 0.119 g , were utilized. Five groups of quail, each with replicates of 12 birds, were selected at random. The cages used to house the birds were standard cages; the water and feed were supplied ad libitum. The fifth week of age was the end point of this study. The following were the experimental groups: the first group was fed the basal diet without any supplementation (0 $g\;{\mathrm{kg}}^{-1}$ ), the second group was fed the basal diet and 0.4 $g\;\mathrm{BPO}\;{\mathrm{kg}}^{-1}$ , the third group was fed the basal diet and 0.8 $g\;\mathrm{BPO}\;{\mathrm{kg}}^{-1}$ , the fourth group was fed the basal diet and 1.2 $g\;\mathrm{BPO}\;{\mathrm{kg}}^{-1}$ , and the fifth group was fed the basal diet and 1.6 $g\;\mathrm{BPO}\;{\mathrm{kg}}^{-1}$ . The BPO was supplied by El Hawag Natural Oils, Cairo, Egypt. The recommendations of the National Research Council (NRC, 1994) were used to formulate the basal diet (Table 1) to meet the nutrient requirements of the birds. Throughout the trial, quails were exposed to light for 24 h .

**Table 1:** Ingredients and nutrient contents of basal diet of growing Japanese quail. ME: metabolizable energy. TSAA: total sulfur amino acids.

Item	(%)
Ingredient (%)	
Maize 8.5 %	51.80
Soybean meal 44 %	36.70
Maize gluten meal 62 %	5.21
Soybean oil	2.90
Limestone	0.70
Dicalcium phosphate	1.65
Salt	0.30
Premix ${}^{1}$	0.30
L-Lysine	0.13
DL-Methionine	0.11
Choline chloride (50 %)	0.20
Total	100
Calculated composition ${}^{2}$ , %	
ME, $\mathrm{kcal}\;{\mathrm{kg}}^{-1}$	2995
Crude protein	24.00
Calcium	0.80
Nonphytate P	0.45
Lysine	1.30
TSAA	0.92

${}^{1}$ Per kg of diet, the following is provided: vitamin A, 12 000 IU; vitamin D3, 5000 IU; vitamin E, 130.0 mg ; vitamin K3, 3.605 mg ; vitamin B1 (thiamin), 3.0 mg ; vitamin B2 (riboflavin), 8.0 mg ; vitamin B6, 4.950 mg ; vitamin B12, 17.0 mg ; niacin, 60.0 mg ; D-biotin, 200.0 mg ; calcium D-pantothenate, 18.333 mg ; folic acid, 2.083 mg ; manganese, 100.0 mg ; iron, 80.0 mg ; zinc, 80.0 mg ; copper, 8.0 mg ; iodine, 2.0 mg ; cobalt, 500.0 mg ; and selenium, 150.0 mg . ${}^{2}$ Calculated according to NRC (1994).

### 2.2 Growth performance

At 1, 3, and 5 weeks of age, each individual's body weight (BW) was measured. The body weight gain (BWG) was also measured over the course of the experiments. Moreover, feed intake was continuously measured during the trial periods on a replicated basis to estimate the feed conversion ratio (FCR; given in units of gfeedperggain ).

### 2.3 Carcass traits

A total of five quails per replicate (12 per group) were randomly chosen, weighed, and manually slaughtered for carcass examination at the end of the experiment (42 d old). Carcass weight, giblets, gizzard, heart, liver, and intestines were weighed and reported as percentages of the slaughter weight. Intestinal pH was also measured.

### 2.4 Microbiological analysis

In order to determine the total microbial population in the content of the caecum, five quail per replicate (12 per group) were chosen at random and slaughtered at the final of the experiment (5 weeks). Fresh samples of caecal digesta (1 gperquail ) were put in 250 mL conical flasks with 90 mL of saline solution made up of 0.1 % peptone and 0.85 % NaCl . Following thorough mixing, the liquid was serially diluted up to 106 times. MacConkey agar medium was used to count all coliforms. *E. coli* was identified using biochemical techniques such the citrate reactions, indole test, Voges-Proskauer and methyl red. *Salmonella* spp. were counted using Salmonella Shigella agar (SSA) media (Oxoid CM 99). *Salmonella* spp. were present as evidenced by the formation of black colonies on SSA. The colony-forming unit, $\mathrm{CFU}\;g^{-1}$ , was used for all the obtained microbiological results (Xia et al., 2004).

### 2.5 Blood parameters

The blood was randomly obtained from five birds per treatment after slaughter and placed in sterilized tubes with rubber stoppers. Plasma samples were collected from the samples of the blood and held at - 20 °C for analysis after being centrifuged for 15 min at 2147 × g . By utilizing kits from Bio Diagnostic (Giza, Egypt), the following parameters were analyzed spectrophotometrically: total protein, albumin, aspartate transaminase (AST), alanine transaminase (ALT), triglycerides, total cholesterol, high-density lipoprotein (HDL) cholesterol, and low-density lipoprotein (LDL) cholesterol levels. Kits produced by Spectrum Diagnostics (Cairo, Egypt) were used to estimate the values of immunoglobulins G (IgG), M (IgM), and A (IgA). By utilizing kits and a spectrophotometer (Shimadzu, Japan), the levels of catalase (CAT), superoxide dismutase (SOD), and malondialdehyde (MDA) activity and reduced glutathione (GSH) activity were evaluated in plasma.

### 2.6 Statistical analysis

All of the trial's statistical analyses were carried out using SAS (SAS Institute Inc., 2001). For growth performance metrics, the pen was the research unit; however, for all other parameters, it was the individual bird. Using one-way ANOVA and Tukey's test, the growth performance, carcass traits, serum components, and antioxidant indices were investigated. At P < 0.05, significance was concluded. The statistical model is $Y_{ij}$ = $T_{i}+e_{ij}$ , where $Y_{ij}$ is the observation and overall mean, $T_{i}$ is the treatment effect, and $e_{ij}$ is the random error.

**Table 2:** Growth performance of growing Japanese quail as affected by dietary treatments during the experiment.

Items	Black pepper oil level ( $g\;{\mathrm{kg}}^{-1}$ )					SEM	P value
	0	0.4	0.8	1.2	1.6		
Body weight ( g )							
1 week	29.60	29.67	29.64	29.69	29.67	0.119	0.9895
3 weeks	109.11 ${}^{d}$	117.33 ${}^{c}$	121.78 ${}^{b}$	116.89 ${}^{c}$	125.00 ${}^{a}$	0.876	< 0.0001
5 weeks	191.84 ${}^{d}$	204.11 ${}^{\text{bc}}$	212.33 ${}^{a}$	201.67 ${}^{c}$	207.56 ${}^{\text{ab}}$	1.550	< 0.0001
Body weight gain ( $g\;d^{-1}$ )							
1–3 weeks	5.68 ${}^{c}$	6.26 ${}^{b}$	6.58 ${}^{a}$	6.23 ${}^{b}$	6.81 ${}^{a}$	0.065	< 0.0001
3–5 weeks	5.91	6.20	6.47	6.06	5.90	0.132	0.1568
1–5 weeks	5.79 ${}^{d}$	6.23 ${}^{\text{bc}}$	6.52 ${}^{a}$	6.14 ${}^{c}$	6.35 ${}^{\text{ab}}$	0.055	< 0.0001
Feed intake ( $g\;d^{-1}$ )							
1–3 weeks	14.35 ${}^{a}$	12.45 ${}^{\text{bc}}$	11.27 ${}^{c}$	12.65 ${}^{b}$	12.12 ${}^{\text{bc}}$	0.310	0.0044
3–5 weeks	24.42	23.66	24.5	25.15	24.33	0.523	0.5885
1–5 weeks	19.38 ${}^{a}$	18.05 ${}^{c}$	17.89 ${}^{c}$	18.90 ${}^{\text{ab}}$	18.23 ${}^{\text{bc}}$	0.226	0.0051
Feed conversion ratio ( gfeedperggain )							
1–3 weeks	2.53 ${}^{a}$	1.99 ${}^{b}$	1.71 ${}^{c}$	2.03 ${}^{b}$	1.78 ${}^{c}$	0.051	< 0.0001
3–5 weeks	4.14 ${}^{a}$	3.82 ${}^{b}$	3.79 ${}^{b}$	4.15 ${}^{a}$	4.13 ${}^{a}$	0.084	0.0410
1–5 weeks	3.35 ${}^{a}$	2.90 ${}^{c}$	2.74 ${}^{d}$	3.08 ${}^{b}$	2.87 ${}^{c}$	0.025	< 0.0001

Means in a row with different superscripts differ significantly.

## 3 Results

### 3.1 Growth performance

As shown in Table 2, dietary supplementation of BPO in the growing-quail diet caused appreciable variations in growth performance parameters. The group supplemented with BPO at a level of 0.8 $g\;{\mathrm{kg}}^{-1}$ revealed a significant increase ( p < 0.05) in final body weight (212.33 g ) and body weight gain (6.52 $g\;d^{-1}$ ) when compared with the control. Moreover, the group whose diet was supplemented with BPO at a level of 0.8 $g\;{\mathrm{kg}}^{-1}$ from 1–5 weeks significantly consumed less feed (17.89 $g\;d^{-1}$ ) than the other groups and the control. The FCR of the quails that were fed BPO was higher than that of the control quails between the ages of 1 and 3 weeks. The best values for FCR ( P < 0.0001) were found in the 3–5-week and 1–5-week age groups whose diets were supplemented with 0.8 gBPO in comparison to other BPO-supplemented groups and the control.

**Table 3:** Carcass traits and relative organs of growing Japanese quail as affected by dietary treatments during the experiment.

Items	Black pepper oil level ( $g\;{\mathrm{kg}}^{-1}$ )					SEM	P value
	0	0.4	0.8	1.2	1.6		
Carcass (%)	74.04	77.11	76.87	78.88	75.76	1.599	0.3779
Liver (%)	3.13	2.35	2.98	2.23	2.27	0.390	0.5037
Gizzard (%)	1.86	1.80	1.98	1.88	1.90	0.117	0.9175
Heart (%)	1.08	0.98	0.93	1.04	1.01	0.051	0.4368
Intestine (%)	3.87	4.21	4.31	4.07	4.24	0.268	0.8226
Giblets (%)	6.22	5.13	5.89	5.15	5.18	0.453	0.5979
Dressing (%)	80.26	82.25	82.76	84.03	80.95	1.279	0.3613
Caecal content pH	6.94 ${}^{a}$	6.84 ${}^{a}$	6.26 ${}^{c}$	6.47 ${}^{\text{bc}}$	6.71 ${}^{\text{ab}}$	0.085	0.0019

Means in a row with different superscripts differ significantly.

### 3.2 Carcass traits

As shown in Table 3, dietary BPO supplementation had no significant impact ( P > 0.05) on the ratios of the carcass, dressing, liver, heart, gizzard, giblets, and intestine, for the weight of pre-slaughter quails. In comparison to the other dietary group, the caecal content pH decreased when BPO (0.8 $g\;{\mathrm{kg}}^{-1}$ ) was added to quail diets.

**Table 4:** Effects of treatments on caecal microbiota (total bacterial count, lactobacilli count, coliform, *E. coli*, and *Salmonella* spp.) in growing Japanese quail.

Items	Black pepper oil level ( $g\;{\mathrm{kg}}^{-1}$ )					SEM	P value
	0.0	0.4	0.8	1.2	1.6		
Microbiological count (log $\mathrm{CFU}\;g^{-1}$ )							
Total bacterial count	8.67 ${}^{a}$	8.12 ${}^{c}$	8.65 ${}^{a}$	8.57 ${}^{a}$	8.28 ${}^{b}$	0.030	< 0.0001
Lactobacilli	7.66 ${}^{a}$	6.85 ${}^{d}$	7.55 ${}^{a}$	7.40 ${}^{b}$	7.12 ${}^{c}$	0.031	< 0.0001
Coliform	4.45 ${}^{a}$	3.42 ${}^{\text{bc}}$	3.51 ${}^{b}$	3.34 ${}^{c}$	3.32 ${}^{c}$	0.048	< 0.0001
*E. coli*	4.22 ${}^{a}$	3.10 ${}^{c}$	3.85 ${}^{b}$	3.96 ${}^{b}$	3.16 ${}^{c}$	0.049	< 0.0001
*Salmonella*	4.51 ${}^{a}$	2.26 ${}^{e}$	3.25 ${}^{b}$	3.11 ${}^{c}$	2.47 ${}^{d}$	0.039	< 0.0001

Means in a row with different superscripts differ significantly.

### 3.3 Microbiological analysis

Table 4 lists caecal content microbiomes in the growing quail tested. In comparison to the control, quails' diets supplemented with BPO (0.4, 0.8, 1.2, and 1.6 $g\;{\mathrm{kg}}^{-1}$ ) had significant ( P < 0.05) decreased levels of total bacterial count (TBC), lactobacilli, *E. coli*, coliform, and *Salmonella* colonization.

**Table 5:** Blood protein metabolite, liver, and kidney functions of growing Japanese quail as affected by dietary treatments during the experiment.

Items ${}^{1}$	Black pepper oil level ( $g\;{\mathrm{kg}}^{-1}$ )					SEM	P value
	0	0.4	0.8	1.2	1.6		
Total protein ( $g\;{\mathrm{dL}}^{-1}$ )	2.57 ${}^{c}$	3.05 ${}^{b}$	3.49 ${}^{\text{ab}}$	3.28 ${}^{\text{ab}}$	3.58 ${}^{a}$	0.108	0.0034
Albumin ( $g\;{\mathrm{dL}}^{-1}$ )	1.54 ${}^{b}$	1.86 ${}^{a}$	1.99 ${}^{a}$	1.88 ${}^{a}$	1.86 ${}^{a}$	0.069	0.0180
Globulin ( $g\;{\mathrm{dL}}^{-1}$ )	1.03 ${}^{d}$	1.19 ${}^{\text{cd}}$	1.50 ${}^{\text{ab}}$	1.40 ${}^{\text{bc}}$	1.72 ${}^{a}$	0.063	0.0014
A/G ratio	1.54 ${}^{a}$	1.57 ${}^{a}$	1.33 ${}^{\text{ab}}$	1.35 ${}^{\text{ab}}$	1.08 ${}^{b}$	0.069	0.0215
AST ( $\mathrm{IU}\;L^{-1}$ )	174.43 ${}^{c}$	269.93 ${}^{a}$	250.13 ${}^{a}$	199.17 ${}^{\text{bc}}$	231.47 ${}^{\text{ab}}$	12.002	0.0039
ALT ( $\mathrm{IU}\;L^{-1}$ )	10.10 ${}^{\text{cd}}$	12.84 ${}^{b}$	18.29 ${}^{a}$	10.79 ${}^{c}$	8.29 ${}^{d}$	0.562	< 0.0001
Creatinine ( $\mathrm{mg}\;{\mathrm{dL}}^{-1}$ )	0.46 ${}^{a}$	0.31 ${}^{b}$	0.48 ${}^{a}$	0.51 ${}^{a}$	0.42 ${}^{\text{ab}}$	0.035	0.0430
Urea ( $\mathrm{mg}\;{\mathrm{dL}}^{-1}$ )	1.61 ${}^{a}$	1.36 ${}^{\text{ab}}$	1.36 ${}^{\text{ab}}$	1.18 ${}^{b}$	1.24 ${}^{b}$	0.074	0.0275

Means in a row with different superscripts differ significantly. ${}^{1}$ ALT: alanine aminotransferase. AST: aspartate aminotransferase. A/G : albumin/globulin ratio.

**Table 6:** Lipid profile of growing Japanese quail as affected by dietary treatments during the experiment.

Items ${}^{1}$	Black pepper oil level ( $g\;{\mathrm{kg}}^{-1}$ )					SEM	P value
	0	0.4	0.8	1.2	1.6		
Total cholesterol ( $\mathrm{mg}\;{\mathrm{dL}}^{-1}$ )	580.81 ${}^{a}$	281.24 ${}^{c}$	372.43 ${}^{b}$	196.19 ${}^{d}$	234.11 ${}^{\text{cd}}$	13.861	< 0.0001
Triglycerides ( $\mathrm{mg}\;{\mathrm{dL}}^{-1}$ )	349.53 ${}^{a}$	92.39 ${}^{c}$	225.25 ${}^{b}$	196.29 ${}^{b}$	101.73 ${}^{c}$	10.362	< 0.0001
HDL ( $\mathrm{mg}\;{\mathrm{dL}}^{-1}$ )	29.91 ${}^{b}$	45.81 ${}^{a}$	48.22 ${}^{a}$	44.58 ${}^{a}$	52.87 ${}^{a}$	3.316	0.0085
LDL ( $\mathrm{mg}\;{\mathrm{dL}}^{-1}$ )	480.99 ${}^{a}$	216.96 ${}^{c}$	279.16 ${}^{b}$	112.35 ${}^{e}$	160.89 ${}^{d}$	11.878	< 0.0001
VLDL ( $\mathrm{mg}\;{\mathrm{dL}}^{-1}$ )	69.91 ${}^{a}$	18.48 ${}^{c}$	45.05 ${}^{b}$	39.26 ${}^{b}$	20.35 ${}^{c}$	2.072	< 0.0001

Means in a row with different superscripts differ significantly. ${}^{1}$ HDL: high-density lipoprotein. LDL: low-density lipoprotein. VLDL: very low density lipoprotein.

**Table 7:** Antioxidant and immunological parameters of growing Japanese quail as affected by dietary treatments during the experiment.

Items ${}^{1}$	Black pepper oil level ( $g\;{\mathrm{kg}}^{-1}$ )					SEM	P value
	0.0	0.4	0.8	1.2	1.6		
SOD ( $U\;{\mathrm{mL}}^{-1}$ )	0.12 ${}^{b}$	0.25 ${}^{a}$	0.18 ${}^{\text{ab}}$	0.26 ${}^{a}$	0.23 ${}^{a}$	0.021	0.0113
MDA ( $\mathrm{nmol}\;{\mathrm{mL}}^{-1}$ )	0.57 ${}^{a}$	0.27 ${}^{\text{bc}}$	0.32 ${}^{b}$	0.23 ${}^{c}$	0.22 ${}^{c}$	0.026	< 0.0001
CAT ( $\mathrm{ng}\;{\mathrm{dL}}^{-1}$ )	0.12 ${}^{c}$	0.24 ${}^{\text{ab}}$	0.18 ${}^{\text{bc}}$	0.26 ${}^{a}$	0.23 ${}^{\text{ab}}$	0.017	0.0083
GSH ( $\mathrm{ng}\;{\mathrm{mL}}^{-1}$ )	0.13 ${}^{c}$	0.25 ${}^{\text{ab}}$	0.20 ${}^{b}$	0.27 ${}^{a}$	0.25 ${}^{\text{ab}}$	0.015	0.0024
IgG ( $\mathrm{mg}\;{\mathrm{dL}}^{-1}$ )	0.24 ${}^{d}$	0.44 ${}^{\text{bc}}$	0.39 ${}^{c}$	0.51 ${}^{\text{ab}}$	0.56 ${}^{a}$	0.029	0.0004
IgA ( $\mathrm{mg}\;{\mathrm{dL}}^{-1}$ )	0.64 ${}^{a}$	0.40 ${}^{\text{bc}}$	0.43 ${}^{b}$	0.35 ${}^{\text{bc}}$	0.33 ${}^{c}$	0.027	< 0.0001
IgM ( $\mathrm{mg}\;{\mathrm{dL}}^{-1}$ )	0.22 ${}^{c}$	0.42 ${}^{b}$	0.38 ${}^{b}$	0.49 ${}^{\text{ab}}$	0.58 ${}^{a}$	0.034	0.0009

Means in a row with different superscripts differ significantly. ${}^{1}$ SOD: superoxide dismutase. MDA: malondialdehyde. CAT: catalase. GSH: reduced glutathione. IgG: immunoglobulin G. IgA: immunoglobulin A. IgM: immunoglobulin M.

### 3.4 Blood parameters

According to Table 5, adding BPO to the diets in any amount resulted in statistically significant variations in the levels of total protein, albumin, globulin, albumin/globulin ( A/G ) ratio, ALT, and AST ( P < 0.05). Total protein levels were considerably higher in the BPO-supplemented group of 1.6 $g\;{\mathrm{kg}}^{-1}$ in comparison to the control and BPO-supplemented groups of 0.4, 0.8, and 1.2 $g\;{\mathrm{kg}}^{-1}$ ( P = 0.0034). The A/G levels were significantly decreased ( P = 0.0215) in BPO-supplemented groups (0.8, 1.2, and 1.6 $g\;{\mathrm{kg}}^{-1}$ ), but the levels of plasma globulin were significantly increased ( P = 0.0014) in birds fed a BPO-containing diet (0.4, 0.8, 1.2, and 1.6 $g\;{\mathrm{kg}}^{-1}$ ) in comparison to birds in the control. The liver enzyme activity in the plasma (ALT, P < 0.0001, and AST, P = 0.0039) showed a significant increase in the groups whose diets were supplemented with 0.4 and 0.8 gBPO in comparison to the control. The ALT levels tended to be the lowest in the BPO (1.6 $g\;{\mathrm{kg}}^{-1}$ ) group. The addition of BPO to the diets, at any dose, revealed a significant variation in levels of creatinine and urea ( P = 0.0430) and ( P = 0.0275), respectively. The group whose diet was supplemented with BPO at a level of 0.4 g revealed the lowest plasma creatinine level (0.31 $\mathrm{mg}\;{\mathrm{dL}}^{-1}$ ) in comparison to the control and other treated groups. Moreover, the group treated with BPO at a level of 1.2 g showed the lowest plasma urea level (1.18 $\mathrm{mg}\;{\mathrm{dL}}^{-1}$ ) in comparison to the control and other treated groups. According to Table 6, BPO supplementation to the diet reduced plasma total cholesterol levels ( P < 0.0001) and increased plasma HDL levels ( P = 0.0085) in the diet. All BPO-supplemented groups' triglyceride, LDL, and very low density lipoprotein (VLDL) values revealed a significant decrease ( P < 0.05) in comparison to the control. The findings of the plasma's antioxidant status are revealed in Table 7. Concerning the activity of SOD in the plasma, there were variations between treatments that were statistically significant ( P = 0.0113). BPO treatment groups had greater levels of glutathione (0.4, 1.2, and 1.6 $g\;{\mathrm{kg}}^{-1}$ than the control group ( P = 0.0024). BPO (0.4, 1.2, and 1.6 $g\;{\mathrm{kg}}^{-1}$ ) groups had elevated catalase activity compared to the control ( P = 0.0083). The levels of malondialdehyde were decreased ( P < 0.0001) in the groups fed the diet supplemented with BPO (0.4 – 0.8 – 1.2 – 1.6) $g\;{\mathrm{kg}}^{-1}$ in comparison to the control. As indicated in Table 7, the plasma levels of IgG, IgM, and IgA were significantly different in each group ( p < 0.05), and the groups supplemented with BPO (0.4, 0.8, 1.2, and 1.6 $g\;{\mathrm{kg}}^{-1}$ ) revealed a significant elevation in the plasma levels of IgG and IgM and a significant decrease in plasma IgA levels.

## 4 Discussion

According to some reports, BP increases feed intake, increases nutrient digestibility by stimulating digestive enzymes, and prevents pathogen growth in the gastrointestinal tract (GIT) by acting as an antibiotic (Abd El-Hack et al., 2022). It may improve the villi's capacity for absorption to aid in nutrient uptake and assimilation (Pliego et al., 2020). Additionally, it has been discovered that broiler hens that received dietary BP supplements had thicker mucosa and sub-mucosa linings in their duodenum, jejunum, and ileum (Shahverdi et al., 2013) and improved ileal muscular contraction (Pliego et al., 2020). Our work demonstrates that BPO addition has a positive effect on quail growth performance parameters such as body weight, body weight gain, and feed conversion ratio of quail. It has been noted that feeding BP to chickens significantly improved their zootechnical parameters (Galib et al., 2011; Ndelekwute et al., 2015; Ufele et al., 2020). FCR and BWG were improved (2 %–9 %) in broiler chickens fed BP at 2.5, 5.0, 7.5, and 10.0 $g\;{\mathrm{kg}}^{-1}$ (Al-Kassie et al., 2011), which may be attributed to BP's antioxidative action in preventing villi damage from oxidative deterioration (Ashokkumar et al., 2020, 2021). These findings were confirmed by Ufele et al. (2020), who showed that broiler chicken feed intake and BWG were positively impacted by BP supplementation at a rate of 5 $g\;{\mathrm{kg}}^{-1}$ . These developments in zootechnical features are perhaps attributed to the presence of bioactive components in diets based on BP's capacity to boost the expression of digestive enzyme genes and decrease the growth of harmful bacteria in birds' GIT. This might also be explained by the capacity of BP to promote digestive enzymes' release, boost digestion in GIT and utilization, and shorten feed retention time in the small intestine, all of which result in greater body weight gain (Galib et al., 2011). In contrast, Puvaca et al. (2014) found that BP-supplemented diets in poultry at 5 or 10 $g\;{\mathrm{kg}}^{-1}$ for 42 d had no impact on BWG or FCR in comparison to the control. For broiler chickens in the last stage of the fattening period fed 7.5 or 10 g of BP seeds per kilogram of feed, a decrease in BWG and FCR was reported (Ndelekwute et al., 2015), suggesting that chickens' growth is negatively impacted by eating an excess of BP seeds. This result validates the findings of Akbarian et al. (2012), who found that male broilers' diets supplemented with 5 $g\;\mathrm{BP}\;{\mathrm{kg}}^{-1}$ exhibited no difference in BWG in comparison to the control. These differences in the performance characteristics of chickens' diets supplemented with BP may be related to the age of the chicken or the level of supplementation. The impact of BP-based diets on broilers' carcass traits and organ weight has been studied by several researchers (Galib et al., 2011; Ghaedi et al., 2014; Al-Kassie et al., 2011; Rahimian et al., 2016). The results of this research, however, have not always been dependable, but they do agree with the findings of Abou-Elkhair et al. (2014), who reported that broilers' diets supplemented with 5 $g\;\mathrm{BP}\;{\mathrm{kg}}^{-1}$ showed no differences in the weights of the thymus or the bursa of Fabricius, gizzard, liver, heart, proventriculus, spleen, or carcass yield. Likewise, Galib et al. (2011) found that supplementing the broiler ration with BP at levels of 10 $g\;{\mathrm{kg}}^{-1}$ had no appreciable impact on the relative organ weights. Also, our result agrees with Al-Kassie et al. (2012), who stated that the organ weight or carcass of broiler chickens was not significantly affected by a blend of black pepper and red pepper powder. The main place for digestive processes and the absorption of nutrients is the small intestinal tract (Abdelnour et al., 2019). According to the results of the current study, adding BPO to the diet of growing quails decreased caecal content pH. According to Jamroz et al. (2006), phytogenic feed additives may improve gut health because they modify the intestinal contents' pH and promote mucus secretion in birds, which reduces pathogen adherence. Furthermore, numerous in vivo investigations have shown that black pepper essential oil has strong antibacterial properties against dangerous strains of *Staphylococcus* and *Escherichia coli* (Zhang et al., 2017; Abdallah and Abdalla, 2018). According to certain research done on Japanese quails (Mehri et al., 2015) and broiler chickens (Chowdhury et al., 2018), various polyunsaturated fatty acids (PFAs; peppermint and cinnamon) have an antibacterial effect in vivo against intestinal tract pathogenic microorganisms including coliform and *E. coli*. For all examined levels, birds fed BPO-enriched diets had lower levels of *Salmonella*, coliform, TBC, lactobacilli, and *E. coli* colonization in comparison to the control. It has been established that BP increases the growth of beneficial gut microorganisms such as *Lactobacillus* (Ghaedi et al., 2014; Kishawy et al., 2022). Moreover, BP reduced the development of harmful microorganisms in the chicken gut (Naidu et al., 1999).

By supplying birds with herbal products, one can alter the blood's biochemical profile (Moharreri et al., 2022). Higher plasma protein concentration levels correlate to higher dietary protein quality (Ogbuewu et al., 2015). In birds, Akinfola et al. (2007) discovered a favorable link between protein quality intake and the levels of total plasma proteins. Furthermore, the supplementation of 0.5 $g\;\mathrm{BPO}\;{\mathrm{kg}}^{-1}$ of broiler chickens boosted plasma protein levels (Kishawy et al., 2022), indicating that BP supplementation did not impact the quality of protein in the diet. The current results of total protein and globulin in plasma showed a significant increase. According to earlier research, Tayeb et al. (2015) reported that quail given red pepper powder (5 $g\;{\mathrm{kg}}^{-1}$ ) at 49 d of age had significantly lower albumin levels than the control group; however, there was no significant difference in globulin levels. Akinfola et al. (2007) reported that globulin is required for the development of immunoglobulin, and so elevated globulin levels in birds supplemented with BP in diets imply an improved resistance to infection. Other investigations discovered that BP had no effect on total protein or albumin levels in plasma in broiler chickens (Abou-Elkhair et al., 2014; Sugiharto et al., 2021). Several studies stated no significant influence of BP on ALT and AST levels in plasma in broiler chickens (Abou-Elkhair et al., 2014; Kishawy et al., 2022; Sugiharto et al., 2021), indicating that BP has a hepatoprotective effect in poultry. Broilers fed BP essential oils may have a non-significant elevation in both ALT and AST levels due to BP's hepatoprotective capacities, as Damanhouri and Ahmad (2014) discovered that supplementing BP in broilers' diets enhanced liver activity. The renal function is evaluated via serum creatinine levels, and a rise in serum creatinine indicates impaired kidney function (Aslam et al., 2010). In the present study, there were significant differences in creatinine and urea levels when BPO was added to the quails' diets ( P < 0.05). In addition, the group treated with BPO at a level of 0.4 g had the lowest plasma creatinine level (0.31 $\mathrm{mg}\;{\mathrm{dL}}^{-1}$ ) in comparison to the control; additionally, the group supplemented with BPO at a level of 1.2 g had the lowest plasma urea level (1.18 $\mathrm{mg}\;{\mathrm{dL}}^{-1}$ ) in comparison to the control and other treated groups, indicating that BPO is safe for renal function.

Serum cholesterol and triglyceride concentrations can represent fat metabolism (Sharifi et al., 2023). The present studies found that the group supplemented with BPO had decreased serum cholesterol and triglyceride concentrations. HDL is required for fat transport and distribution to tissues and cells. Low levels of BP (5 $g\;{\mathrm{kg}}^{-1}$ ) are stated to produce moderate changes in the metabolism of cholesterol by boosting the cholesterol 7 alpha-hydroxylase enzyme activity (Ramesh et al.1996). Our findings revealed that BPO supplementation in the diet improved the concentrations of HDL and decreased the concentrations of LDL and VLDL. Our result is similar to other authors' observations, who reported that supplementation of BP in the diet of chickens reduced lipid and cholesterol levels (Ghaedi et al., 2014; Shahverdi et al., 2013; Kishawy et al., 2022; Akbarian et al., 2012). This result may be attributed to BP's reduction of acetyl-CoA production, which is required for fat metabolism. Studies on animals have demonstrated that the bioactive components of BP, especially piperine, have antiperoxidative effects (Dhuley et al., 1993). Because of this, broiler hens' diets supplemented with BP caused lower levels of plasma cholesterol and LDL cholesterol (Galib et al., 2011; Ghaedi et al., 2014; Shahverdi et al., 2013; Puvaca et al., 2014), which could be attributed to BP antioxidative properties. Ashokkumar et al. (2020) and Lee et al. (2020) also discovered that incorporating BP into the diet significantly elevates blood HDL cholesterol concentrations. Antioxidants are substances, either natural or artificial, that can be utilized to decrease the risk of degenerative and chronic diseases by scavenging free radicals and preventing their formation (Halliwell, 2000). The significant activity of CAT and GSH in groups that were fed BPO, as well as the low levels of MDA, confirms that it is an effective antioxidant that can neutralize free radicals. Piperine possesses potential antioxidant action and anti-lipid peroxidation in rats supplemented with a high-fat diet, which results in oxidative stress in cells (Vijayakumar et al., 2004). Our findings showed that dietary supplementation with BPO elevated the concentrations of SOD, CAT, and GSH and decreased the concentrations of MDA. Our results agree with Jeena et al. (2014), who discovered that BPO removes superoxides and reduced tissue fat peroxidation in vitro. Herbal preparations boost immunological responses by stimulating the creation and release of immunoglobulins, lymphocytes, and interferon- γ (Barnes et al., 1972; Faramarzi et al., 2013). The present result shows that BPO supplementation to quails' diet increased the levels of immunoglobulins (IgG and IgM), and these findings agree with Kishawy et al. (2022), who reported that supplementation of 0.25 or 0.5 $g\;\mathrm{BPO}\;{\mathrm{kg}}^{-1}$ in broiler diet improved immunity characteristics, as revealed by elevated lysozyme action, immune system antibody levels, and phagocytic metrics, in comparison with birds fed a diet without supplemental BPO. Moreover, Kishawy et al. (2022) stated that in broiler chickens, BP increases the *IL-10* and *IgA* gene expression.

## 5 Conclusion

It can be proven that BPO supplementation in Japanese quails' diet can boost growth performance and antioxidant indices, enhance lipid profile and carcass traits, and reduce intestinal infections. Hence, BPO supplements may be used to improve the growth performance and health conditions of growing Japanese quails.

## Data Availability

The data presented in this study are available on request from the corresponding author.
